# Translation and validation of the Persian version of the morbid obesity quality of life questionnaire

**DOI:** 10.1186/s13104-025-07602-8

**Published:** 2026-01-03

**Authors:** Shima Ghannadi, Maryam Ganjalikhani, Ali Kordi, Ramin Kordi, Kazem Khalagi, Elham Sharafi, Hanieh-Sadat Ejtahed, Mohammad Hossein Pourgharib Shahi, Shirin Hasani Ranjbar

**Affiliations:** 1 Obesity and Eating Habits Research Center, Endocrinology and Metabolism Clinical Sciences Institute, Tehran University of Medical Sciences, Tehran, Iran; 2 Sports Medicine Research Center, Neuroscience Institute, Tehran University of Medical Sciences, Tehran, Iran; 3 Osteoporosis Research Center, Endocrinology and Metabolism Clinical Sciences Institute, Tehran University of Medical Sciences, Tehran, Iran; 4 Psychosomatic Medicine Research Center, Imam Khomeini Hospital Complex, Tehran University of Medical Sciences, Tehran, Iran

**Keywords:** Quality of life, Obesity, Morbid, Laval questionnaire, Psychometrics, Validation, Persian

## Abstract

**Objective:**

Morbid obesity significantly impairs quality of life, leading to increased morbidity, healthcare costs, and longer hospital stays. Generic quality of life instruments may fail to capture the disease-specific challenges experienced by individuals with morbid obesity. The Laval questionnaire was developed to assess quality of life specifically in this population. This study aimed to translate and culturally adapt the Laval questionnaire into Persian and to evaluate its reliability and validity among Persian-speaking individuals with morbid obesity. The study was conducted at Shariati Hospital, a major bariatric surgery center in Tehran, Iran. Participants aged 19 to 65 years with morbid obesity who were candidates for bariatric surgery based on international criteria were recruited through consecutive sampling.

**Results:**

The Persian version demonstrated excellent content validity, with a CVI greater than 0.9 and a CVR exceeding 0.8. The overall Cronbach’s alpha was 0.896, indicating strong internal consistency. Each domain showed a Cronbach’s alpha above 0.8, except for the sex life domain, which showed moderate consistency. All ICC values were statistically significant (*p* < 0.001), confirming excellent test–retest reliability. These findings support the Persian adaptation of the Laval questionnaire as a reliable and valid instrument for assessing quality of life among individuals with morbid obesity in Iran.

**Supplementary Information:**

The online version contains supplementary material available at10.1186/s13104-025-07602-8.

## Introduction

Obesity and its complications are among the most serious health problems in both developed and developing countries [1]. Its prevalence has increased significantly worldwide in recent decades [2]. In Iran, the number of obesity cases increased from 2 million in 1980 to 11 million in 2015 [3]. A recent study by Pourfarzi reported a prevalence of obesity of 45% in the Iranian population in 2022 [4]. Obesity in various classes poses a serious health burden to the population [5]. Among the different types of obesity, the greatest health problems and economic burden are associated with the upper end of the body mass index (BMI) distribution, that is, morbid obesity [6]. Morbid obesity is defined as a body mass index (BMI) greater than 40 kg/m² and is strongly linked to type 2 diabetes, dyslipidemia, cardiovascular disease, depression, and several cancers [7, 8]. Individuals with morbid obesity face more complex health issues and greater challenges within healthcare systems compared to those with moderate obesity [9]. The condition has significant negative consequences, including reduced life expectancy by up to 10 years, decreased quality of life, and increased healthcare costs and hospitalization duration [3, 6].

Diet, exercise, behavioral therapy, pharmacotherapy, and surgery are recognized treatment strategies for morbid obesity [10]. Measuring quality of life (QOL) in these patients is essential to evaluate treatment effectiveness and clinical outcomes [11]. QOL assessment also informs the development of clinical pathways, service delivery models, healthcare policies, and cost-effectiveness analyses [12]. According to the World Health Organization (WHO), QOL reflects an individual’s perception of their position in life within the context of cultural values and social systems [13].

Although general instruments such as the Short Form-36 (SF-36) can be used to assess QOL, they are not designed to capture disease-specific challenges [14,15]. A systematic review based on COSMIN criteria (Obesity Reviews, 2018) classified the Laval Questionnaire as Level C—indicating moderate evidence and emphasizing the need for further cross-cultural validation [16, 17]. This underscores the importance of conducting validation studies in different populations and languages to enhance its psychometric evidence and global applicability. The limited validation data outside the Canadian context further highlight the necessity of such work.

Originally, the Laval Questionnaire consists of 44 items divided into six dimensions: symptoms, activity/mobility, personal hygiene/clothing, emotions, social interaction, and sexual life. Each dimension is rated on a 7-point Likert scale, with higher scores indicating better quality of life. Patients are asked to report how their obesity has affected their lives in the past 4 weeks [18].

Having a validated Persian version of the Laval Questionnaire is crucial to ensure cultural relevance, accurate assessment, and research comparability for Persian-speaking individuals with morbid obesity. The modern Persian language continuum includes Farsi (Iran), Dari (Afghanistan), and Tajik (Tajikistan). The present validation primarily targets Farsi as used in Iran; however, future adaptations may be needed for Dari and Tajik variants due to differences in vocabulary and idiomatic usage. More than 120 million people live in countries where Persian is the official language, and despite the high prevalence of morbid obesity in these populations, no Persian version of the Laval Questionnaire has been reported to date.

Cultural adaptation of a questionnaire requires a careful and systematic approach to achieve linguistic and conceptual equivalence. Therefore, items should be appropriately translated both linguistically and culturally to ensure accurate understanding. This study aimed to translate the Laval Questionnaire and evaluate its reliability and validity among Persian-speaking patients with morbid obesity, thereby providing a standardized tool for clinical research and practice in Iran.

## Materials and methods

### Study design and participants

This cross-sectional study was performed on patients with severe or morbid obesity in Tehran, Iran. The patients were selected using a consecutive sampling method between November 2022 and February 2023. This validation study took place at Shariati Hospital, a prominent center for bariatric surgery in Iran.According to the World Health Organization (WHO), morbid obesity is defined as a BMI ≥ 40 kg/m². However, in line with international bariatric surgery guidelines, individuals with a BMI ≥ 35 kg/m² who have obesity-related comorbidities are also considered eligible candidates for surgical intervention. Therefore, the inclusion criteria in this study comprised adults aged 19–65 years with BMI ≥ 35 kg/m² who were candidates for bariatric surgery according to these clinical standards.Written informed consent was obtained from all participants prior to inclusion. The presence of comorbidities did not lead to exclusion. Patients with cognitive impairments or those not deemed eligible for bariatric surgery were excluded. Recruitment occurred during the patients’ initial visit with the bariatric surgeon, coinciding with the first administration of assessment tools.

### Translation and culture adaptation

The protocol for this study was approved by the ethics committee of the Tehran University of Medical Sciences (IR.TUMS.EMRI.REC.1401.030). Based on standard and internationally accepted guidelines, translation and cultural adaptation were carried out in five specific steps [19, 20].

During the “forward translation” phase, the English version of the LAVAL questionnaire was translated into Persian independently by two translators. This included an expert scientific linguist and a sports medicine specialist who is a native Persian speaker and an expert in English. They recorded all linguistic and cultural concepts and discussed any discrepancies in a meeting. Following consensus, a unified Persian version was reached.

The Persian translation was then back-translated by a native English speaker proficient in Persian and living in Iran (with an understanding of the cultural context) and unaware of the original version, in the next step.

In the third step, the multi-disciplinary committee including ten experts which consisted of an epidemiologist, an endocrinologist, two psychiatrists, two general physicians, two sports medicine specialists, and two nutritionists, reviewed for consistency, and final wording, and consolidated the pre-final Persian version. Moreover, they reviewed the introduction and instruction to the questionnaire as well as reviewed the scaling of responses to each question. Any discrepancies were discussed and resolved to achieve a satisfactory translated version for the pilot study.

The fourth step involved conducting a pilot study to pre-test the Persian version of the questionnaire on 15 patients with morbidly obesity. This was done to assess how respondents process and respond to questionnaire items. The process included participants completing the translated LAVAL questionnaire and then providing feedback on their understanding of individual questions, response options, and instructions, as well as verbalizing how they had produced their answers.

In the final stage, the questionnaire was adjusted to align the scoring weights with the cultural context. Following the completion of the questionnaire each participant, they were individually interviewed to discuss their interpretation of each question and their chosen responses. Additionally, participants were invited to highlight any words or phrases that they found challenging to comprehend and provide feedback on the questionnaire as a whole. As part of evaluating the questionnaire’s face validity, the Misunderstanding Index was computed for each item following the pilot study. This involved asking a group of participants to articulate their understanding of each question. The Misunderstanding Index indicates the proportion of participants whose interpretation differed from the intended meaning of the questions [19]. After reviewing the responses from patients, the expert panel reached a consensus on several additional adjustments. For instance, the term “physical appearance” in question 39 was modified to “body appearance,” and in consideration of religious beliefs and cultural practices, the term “wiping” after using the toilet in question 42 was changed to “washing.” To evaluate content validity, each item in the dataset underwent assessment using the Content Validity Index (CVI) by the expert panel members [21]. Subsequently, the final Persian version of the dataset (as shown in Appendix) was endorsed by the committee, affirming its content and face validity.

### Statistical analysis

In this study, all data analysis was conducted using SPSS 22 (SPSS Inc, Illinois, USA). Quantitative and categorical variables were described as Mean (SD) and number (percent). The test–retest reliability of each dataset question was evaluated using the Intra-class Correlation Coefficient (ICC) based on the One-way random effects model. Internal consistency for each dimension of the dataset was measured using Cronbach’s alpha coefficient to assess homogeneity and reproducibility. Face and content validity were determined through the Misunderstanding Index, Content Validity Index (CVI), and Content Validity Ratio) CVR) respectively [19, 21]. Furthermore, Pearson’s correlation coefficient was used to assess the convergent validity of the total scores of the Persian version of Laval and the Persian version of the IWQlite questionnaire.

### The Laval questionnaire

The Laval Questionnaire, comprising 44 items, serves as an evaluative tool in clinical trials. Originally developed in French [18], the questionnaire’s construction methodology is detailed elsewhere. It consists of 6 domains: symptoms (10 items), activity/mobility (9 items), personal hygiene/clothing (5 items), emotions (11 items), social interactions (7 items), and sexual life (2 items). Each domain is rated on a 7-point Likert scale, with higher scores indicating a better quality of life. Patients are required to assess how their obesity impacted their lives in the past 4 weeks.

### The impact of weight on quality of life (IWQOL-lite)

The IWQOL-lite, a self-administered questionnaire, assesses the impact of weight on quality of life in individuals with obesity [22]. It comprises 31 questions organized into five dimensions: physical function, self-esteem, sexual activity, anxiety in public places, and work. Najjarzadeh et al. translated and culturally adapted the Persian version of it [23].

## Results

### Quantitative content validity indexes of the Laval questionnaire

To assess content validity, ten experts evaluated each item in terms of relevance, clarity, simplicity, and essentiality. Values ranged from 0 to 1, where an Item-CVI > 0.79 indicated acceptable relevance, values between 0.70 and 0.79 suggested the need for revision, and values below 0.70 warranted item elimination [24]. Additionally, a CVR value of at least 0.78 was considered acceptable [25].

As shown in Table 1, expert evaluations confirmed high levels of relevance, clarity, and simplicity across all items. All CVI values exceeded 0.9, and all CVR values were greater than 0.8. CVI values above 0.9 demonstrate strong alignment with the intended construct, as well as high clarity and comprehensibility, supporting the excellent content validity of the questionnaire. The Lawshe method was used to calculate these values, all of which surpassed 0.9, indicating that every item was essential and appropriately designed.

**Table 1:** Content validity indicators of the Laval questionnaire questions

Question	R_cvi^1^	C_cvi^2^	S_cvi^3^	CVR^4^	cvi_total^5^
1	1	0.9	1	1	0.97
2	1	0.9	1	1	0.97
3	1	1	1	1	1.00
4	1	0.9	1	1	0.97
5	1	0.9	1	1	0.97
6	1	1	1	1	1.00
7	1	1	1	0.8	1.00
8	1	1	1	1	1.00
9	1	1	1	1	1.00
10	1	1	1	0.8	1.00
11	1	0.9	1	1	0.97
12	1	1	1	1	1.00
13	0.9	1	0.9	1	0.93
14	1	1	1	1	1.00
15	1	1	1	1	1.00
16	0.9	1	0.9	1	0.93
17	1	1	1	0.8	1.00
18	1	1	1	1	1.00
19	1	0.9	1	1	0.97
20	1	0.9	1	1	0.97
21	1	0.9	1	1	0.97
22	1	1	1	1	1.00
23	1	1	1	1	1.00
24	1	0.9	1	1	0.97
25	1	1	1	1	1.00
26	1	1	1	1	1.00
27	1	1	1	1	1.00
28	0.9	1	0.9	1	0.93
29	1	1	1	1	1.00
30	1	0.9	1	1	0.97
31	1	0.9	1	1	0.97
32	1	1	1	1	1.00
33	1	1	1	1	1.00
34	1	1	1	1	1.00
35	1	1	1	1	1.00
36	1	0.9	1	1	0.97
37	1	1	1	1	1.00
38	1	1	1	1	1.00
39	1	0.9	1	1	0.97
40	1	1	1	1	1.00
41	1	1	1	1	1.00
42	1	0.9	1	1	0.97
43	1	1	1	1	1.00
44	1	1	1	1	1.00

### Reliability of the Laval questionnaire

To evaluate reliability, data were obtained from 62 participants, including 53 females (85.5%) and 9 males (14.5%). Regarding educational level, 12 participants (19.4%) had completed primary or adult school, 32 (51.6%) had finished middle or high school, and 18 (29%) held academic degrees. The majority of participants (75.8%) were married. Table 2 summarizes the demographic characteristics of the sample.

**Table 2:** Demographic characteristics of the participants

Variables	Groups	Frequency	Percent	Valid percent	Cumulative percent
Gender	Female	53	85.5	85.5	85.5
	Male	9	14.5	14.5	100.0
level of education	Primary/adult school	12	19.4	19.4	19.4
	Guidance/high school	32	51.6	51.6	71.0
	University degree	18	29.0	29.0	100.0
Marital Status	Single	15	24.2	24.2	24.2
	Married	47	75.8	75.8	100.0
Total		62	100.0	100.0	100.0

As shown in Table 3, the overall Cronbach’s alpha for the questionnaire was 0.896, indicating strong internal consistency. Cronbach’s alpha values for all domains exceeded 0.8, demonstrating high internal reliability, except for the sex life domain, which exhibited moderate consistency with a value below 0.8.

**Table 3:** The reliability of the Laval questionnaire

Domain	Cronbach’s alpha	Result of test–retest reliability		
		*N* of items	Intraclass correlation	*P* value
Clothing	0.860	5	0.563	< 0.001
Mobility	0.913	9	0.560	< 0.001
Emotion	0.935	11	0.576	< 0.001
Sex life	0.620	2	0.468	< 0.001
Social interaction	0.873	7	0.496	< 0.001
Symptoms	0.847	10	0.367	< 0.001
Total	0.896	6	0.591	< 0.001

Test–retest reliability was evaluated using the intra-class correlation coefficient (ICC) to assess the level of agreement between responses over a 2-week interval. All ICC values were statistically significant (*p* < 0.001), confirming excellent test–retest reliability.

### Face validity

The Persian version of the questionnaire was further evaluated for face validity using the Misunderstanding Index after pilot testing. None of the items exceeded a Misunderstanding Index of 20%. The mean completion time for the questionnaire was 10.38 min.

### Criterion validity

According to Pearson’s correlation analysis, there was a strong negative correlation between the Laval and IWQOL questionnaires (*r* = − 0.891, *p* < 0.001), indicating that higher scores on the Laval questionnaire were associated with lower scores on the IWQOL, confirming strong criterion validity.

## Discussion

The findings of this research demonstrate that the Persian adaptation of the Laval questionnaire is a reliable and valid tool for assessing the quality of life in Iranians with morbid obesity. The study revealed that the Persian version of the Laval questionnaire has high content validity, with a CVI greater than 0.9 and a CVR exceeding 0.8. Additionally, the overall questionnaire displayed a Cronbach’s alpha value of 0.896, indicating strong internal consistency. Each domain of the questionnaire also exhibited a Cronbach’s alpha above 0.8, suggesting high internal reliability, except for the sex life domain, which showed moderate consistency with a value below 0.8. Furthermore, all intra-class correlation coefficients (ICCs) were statistically significant (*p* < 0.001), indicating excellent test–retest reliability.

Previous studies have demonstrated good reliability and validity of translated quality-of-life assessment tools across various linguistic and cultural groups [26]. The current study on the Persian version of the Laval questionnaire aimed to establish similar reliability and validity in the Persian-speaking population. Furthermore, earlier studies have evaluated the responsiveness of translated tools to changes in quality of life over time, such as following bariatric surgery or weight management interventions [11, 27]. The present findings confirm that the Persian Laval questionnaire is responsive to changes in the quality of life among individuals with morbid obesity in Iran.

In a recent systematic review, the psychometric properties of QOL assessment tools in patients with morbid obesity were analyzed using the COSMIN checklist [16]. Eight instruments were evaluated, and despite the lack of a complete and comprehensive tool for this purpose, the Laval questionnaire was recommended for research due to its robust psychometric performance [16]. Six of the eight tools were assessed for responsiveness, a critical factor for determining the effectiveness of clinical interventions. The Laval questionnaire demonstrated superior responsiveness, aligning with the COSMIN definition of responsiveness as the ability of an instrument to detect meaningful changes over time [17].

The Laval questionnaire stands out for its specialized focus on individuals with morbid obesity undergoing bariatric surgery, its tailored domains addressing unique physical and psychosocial challenges, and its patient-centered structure [18]. While other tools may offer broader coverage of quality-of-life dimensions, the Laval questionnaire’s specificity and sensitivity make it particularly suitable for assessing outcomes in the bariatric surgery context. By providing a targeted, comprehensive, and patient-centered assessment, it helps clinicians personalize interventions, monitor progress, support patient education, and improve communication between healthcare providers and patients.

### Limitations

This study has several limitations that should be acknowledged. First, the validation was conducted among speakers of the Iranian variety of modern Persian, which, despite sharing a common Persian root, is verbal and culturally different from Dari (Afghanistan) and Tajik (Tajikistan). These variations may affect the interpretation of the questions and therefore the results may not be fully generalizable to all Persian-speaking populations.

Second, the study used a cross-sectional design that precludes assessing the responsiveness of the instrument to changes over time, such as after bariatric surgery or lifestyle interventions. The sample was obtained through convenience sampling from limited clinical centers, which may introduce selection bias and limit its representation of the broader population with morbid obesity. Although the sample size was sufficient for psychometric analyses, it limited comparisons of subgroups (e.g., gender, age groups, comorbidities).

Third, the Laval questionnaire itself has inherent limitations. Its relatively long format (44 questions) may lead to respondent fatigue, especially among participants with lower literacy levels. Future work may explore shorter or more adapted versions to increase feasibility.

Finally, all data were collected in a self-reported manner, which may introduce recall or response bias. Furthermore, although BMI was used as the primary clinical indicator, recent research emphasizes the importance of metabolic and functional parameters in the classification of obesity. The lack of such multidimensional clinical variables in the present study limits the scope of validity assessment.

### Gaps in the literature and areas for future research

While quality of life assessment tools like the Laval questionnaire have been developed and utilized in individuals with morbid obesity, there is a need for more research on the psychometric properties of these tools in diverse populations and settings. Studies should focus on validating these tools in different cultural, socioeconomic, and demographic groups to ensure their reliability and validity across a wide range of populations. Moreover, there is a lack of longitudinal studies that track quality of life outcomes in individuals with morbid obesity over an extended period. Future research should focus on conducting longitudinal assessments to understand how the quality of life changes evolve post-bariatric surgery and how these changes impact long-term outcomes and well-being. More comparative studies are needed to evaluate the effectiveness and sensitivity of different quality of life assessment tools in individuals with morbid obesity. Research comparing tools like the Laval questionnaire with other generic and obesity-specific measures can provide valuable insights into the strengths and limitations of each tool and help identify the most suitable instruments for this population. Future research should focus on evaluating the impact of interventions, such as psychological support, behavioral therapy, and lifestyle modifications, on improving the quality of life in individuals with morbid obesity. Understanding which interventions are most effective in enhancing quality of life outcomes can guide healthcare providers in developing tailored treatment plans for their patients.

## Conclusion

In conclusion, the Persian adaptation of the Laval questionnaire has been shown to be a reliable and valid tool for assessing the quality of life of people with morbid obesity in Iran. The study highlighted the high content validity, strong internal consistency, and excellent test–retest reliability of the questionnaire. The Laval questionnaire’s specificity, patient-centered approach, and capacity to identify changes in response to treatment make it a valuable instrument for assessing quality of life outcomes in the context of bariatric surgery. By providing targeted and comprehensive assessments, this tool can aid healthcare providers in delivering personalized care, monitoring progress, and enhancing communication with patients. Future research should focus on validating quality of life assessment tools in diverse populations, conducting longitudinal studies, and comparing different tools to identify the most effective interventions for improving the quality of life in individuals with morbid obesity.

## Supplementary Information

Below is the link to the electronic supplementary material.

Supplementary Material 1.

## Data Availability

The datasets generated and/or analyzed during the current study are available from the corresponding author on reasonable request.

## Abbreviations

CVI: Content Validity Index
CVR: Content Validity Ratio
ICC: Intra-class correlation coefficient
