# Supplementary material for: Translation and validation of the Persian version of the morbid obesity quality of life questionnaire
Source: BMC Res Notes. 2026 Jan 3;19:48. doi: 10.1186/s13104-025-07602-8 (PMC12866052; doi:10.1186/s13104-025-07602-8)
Supplement: Supplementary file 1 — Supplementary Material 1. [file 13104_2025_7602_MOESM1_ESM.pdf]

پرسشنامه کیفیت زندگی لاوال

نام و نام خانوادگی: .... **تاریخ تولد:** ...../......../.......... **وزن:**........ **قد:**....... **BMI:**..........

تحصیلات: .... **وضعیت تاهل:**................. **جنس:**............. **کدملی:**...............................

هدف این پرسشنامه این است که بفهمیم طی ۴ هفته ی گذشته اضافه وزن شما چطور بر وضعیت زندگیتان تاثیر گذاشته است. از شما در رابطه با تاثیری که اضافه وزن روی فعالیت های روزانه، عواطف، روابط اجتماعی، فعالیت های جنسی، و بهداشت فردی تان داشته است و علایمی که به دنبال چاقی رخ می دهد، سوال پرسیده می شود.

طی ۴ هفته ی گذشته:	همیشه	تقریبا همیشه	بیشتر اوقات	اغلب	گاهی	به ندرت	هرگز
۱. آیا فعالیت های معمول، دچار تنگی نفس می شدید؟							
۲. آیا احساس خستگی می کردید؟							
۳. آیا چندین بار طی شب بیدار می شدید؟							
۴. آیا مواقعی بوده است که به شدت عرق کنید؟							
۵. آیا احساس استرس یا اضطراب داشته اید؟							
۶. آیا احساس می کردید نمی توانید کارهایی که دوست داشتید را انجام دهید؟							
۷. آیا در طی روزمجبور بودید که مرتبا استراحت کنید؟							
۸. آیا از اماکن عمومی و فعالیت های اجتماعی دوری می کردید؟ (سینما، رستوران، مراکز خرید، اتوبوس، ملاقات با دوستان)							
۹. آیا احساس می کردید توسط دیگران پذیرفته نمی شوید؟							
۱۰. . آیا احساس میکردید مانع از، خوب زندگی کردن افراد خانواده تان، همسر، فرزندان یا دوستان می شوید؟							
۱۱. آیا احساس می کردید با دیگران متفاوت هستید؟							
۱۲. آیا احساس می کردید از لحاظ ظاهری (بدنی) جذاب نیستید؟							
۱۳. آیا از نگاه کردن به خودتان در آینه خودداری میکردید؟							
۱۴. آیا لباس هایی می پوشیدید که ظاهرتان را بپوشاند؟							

پرسشنامه کیفیت زندگی لاوال

طی ۴ هفته ی گذشته:	همیشه	تقریبا همیشه	بیشتر اوقات	اغلب	گاهی	به ندرت	هرگز
۱۵. آیا نگران سلامتی خود بوده اید؟							
۱۶. آیا لحظاتی وجود داشت که احساس کنید کنترلی روی اتفاقاتی که برای شما می افتد، ندارید؟							
۱۷. آیا احساس دلسردی و یا افسردگی میکردید؟							
۱۸. آیا احساس میکردید که فقط زنده هستید به جای اینکه بخوبی زندگی کنید؟							
۱۹. آیا لحظاتی بوده است که حس کنید هیچ کاری انجام نمی دهید؟							

طی ۴ هفته ی گذشته:	خیلی زیاد	زیاد	متوسط تا زیاد	متوسط	کم تا متوسط	کم	اصلا
۲۰. آیا کمردرد داشته اید؟							
۲۱. آیا طی روز باید برای بیدار ماندن بجنگید؟							
۲۲. آیا احساس درد در زانو، مچ پا و یا پاهایتان داشتید؟							
۲۳. آیا بین ران ها، شکم، کشاله یا زیر سینه ها زخم یا عرق سوز داشته اید؟							
۲۴. آیا پاهایتان ورم کرده بودند؟							
۲۵. آیا خروپفتان کسی را اذیت می کرد؟							
۲۶. آیا هنگام بستن بند کفش، نشستن یا خم شدن برای برداشتن چیزی مشکلی داشته اید؟							
۲۷. آیا در بالا یا پایین رفتن از پله ها مشکل داشته اید؟							
۲۸. آیا در بلند شدن از صندلی مشکل داشته اید؟							
۲۹. آیا هنگام پوشیدن یا در آوردن لباس (مثل جوراب یا لباس زیر) مشکلی داشته اید؟							

پرسشنامه کیفیت زندگی لاوال

طی ۴ هفته ی گذشته:	خیلی زیاد	زیاد	متوسط تا زیاد	متوسط	کم تا متوسط	کم	اصلا
۳۰. آیا در حرکت و راه رفتن مشکل داشته اید؟							
۳۱. آیا هنگام مشارکت کردن در فعالیت با همسر، فرزند یا دوستانتان مشکلی داشته اید؟							
۳۲. آیا مشکلی در روی هم انداختن پاهای خود داشتید؟							
۳۳. آیا نگران بودید که در اماکن عمومی صندلی ای که اندازه شما باشد، پیدا نکنید؟							
۳۴. آیا نگران بودید که بقیه درباره ی شما چه فکری میکنند؟							
۳۵. آیا ناامیدی های زیادی داشته اید؟							
۳۶. آیا در زندگیتان احساس خوشحال نبودن میکردید؟							
۳۷. آیا در فعالیت های جنسی مشکل داشته اید؟							
۳۸. آیا اعتماد به نفستان را از دست داده اید؟							
۳۹. آیا از بدن (هیکل) خود ناراضی بوده اید؟							
۴۰. آیا از شرکت در مصاحبه ی کاری می ترسیدید؟							
۴۱. آیا در شست و شو بدن مشکل داشته اید؟							
۴۲. آیا در طهارت بعد از دستشویی مشکل داشته اید؟							
۴۳. آیا در کوتاه کردن ناخن های پا مشکل داشته اید ؟							
۴۴. آیا در پیدا کردن لباس هایی که اندازه تان باشد مشکل داشته اید ؟							
